# Altered functional connectivity of cerebellar networks in first-episode schizophrenia

**DOI:** 10.3389/fncel.2022.1024192

**Published:** 2022-11-11

**Authors:** Sitong Feng, Sisi Zheng, Haoming Zou, Linrui Dong, Hong Zhu, Shanshan Liu, Dan Wang, Yanzhe Ning, Hongxiao Jia

**Affiliations:** ^1^Beijing Key Laboratory of Mental Disorders, National Clinical Research Center for Mental Disorders and National Center for Mental Disorders, Beijing Anding Hospital, Capital Medical University, Beijing, China; ^2^Advanced Innovation Center for Human Brain Protection, Capital Medical University, Beijing, China; ^3^Department of Electronic Engineering, Tsinghua University, Beijing, China; ^4^Inner Mongolia Autonomous Region Mental Health Center, Hohhot, China

**Keywords:** first-episode schizophrenia, cerebellum, brain networks, functional connectivity, positive symptom, negative symptom

## Abstract

**Introduction:**

Abnormalities of the cerebellum have been displayed to be a manifestation of schizophrenia (SCH) which is a detrimental psychiatric disorder. It has been recognized that the cerebellum contributes to motor function, sensorimotor function, cognition, and other brain functions in association with cerebral functions. Multiple studies have observed that abnormal alterations in cerebro-cerebellar functional connectivity (FC) were shown in patients with SCH. However, the FC of cerebellar networks in SCH remains unclear.

**Methods:**

In this study, we explored the FC of cerebellar networks of 45 patients with first-episode SCH and 45 healthy control (HC) subjects by using a defined Yeo 17 network parcellation system. Furthermore, we performed a correlation analysis between cerebellar networks’ FC and positive and negative symptoms in patients with first-episode SCH. Finally, we established the classification model to provide relatively suitable features for patients with first-episode SCH concerning the cerebellar networks.

**Results:**

We found lower between-network FCs between 14 distinct cerebellar network pairs in patients with first-episode SCH, compared to the HCs. Significantly, the between-network FC in N2-N15 was positively associated with positive symptom severity; meanwhile, N4-N15 was negatively associated with negative symptom severity. Besides, our results revealed a satisfactory classification accuracy (79%) of these decreased between-network FCs of cerebellar networks for correctly identifying patients with first-episode SCH.

**Conclusion:**

Conclusively, between-network abnormalities in the cerebellum are closely related to positive and negative symptoms of patients with first-episode SCH. In addition, the classification results suggest that the cerebellar networks can be a potential target for further elucidating the underlying mechanisms in first-episode SCH.

## Introduction

Schizophrenia (SCH), described as a disorder of dysconnectivity, manifests as delusions, hallucinations, and cognitive dysfunctions (Friston, 1998). Neuroimaging studies have suggested that abnormalities of the cerebellum may play a crucial role in SCH (Lynall et al., 2010; Kim et al., 2014; Klauser et al., 2017). Postural sway, sensorimotor synchronization, and prediction mediated by the cerebellum are examples of such structural anomalies contributing to sensorimotor dysfunction (Apthorp et al., 2019; Moussa-Tooks et al., 2019). Importantly, functional dysconnectivity within sensorimotor networks in a resting state has been identified in patients with SCH (Walther et al., 2017). Apart from sensorimotor function involving the cerebellum, it has been detected to contribute to motor function, cognition, and emotion, just like with the cerebrum (De Zeeuw et al., 2021). Regional and functional specificity of the cerebellum should be mentioned. There are three lobes of the cerebellar cortex, namely, the anterior lobe, the posterior lobe, and the flocculonodular lobe (Schmahmann et al., 2007). An activation likelihood estimate meta-analysis of neuroimaging studies has demonstrated that sensorimotor tasks trigger the anterior lobe (lobule V) and adjacent lobule VI, with additional foci in lobule VIII; motor activation locates in VIIIA/B; somatosensory activation is in VIIIB (Stoodley and Schmahmann, 2009).

Even though the cerebellum accounts for 10% of total brain mass, it contains four diverse types of neuronal cells in the cerebellar cortex comprising 80% of brain neurons (Azevedo et al., 2009). Furthermore, the cerebellum of humans is functionally coupled to cerebral functional networks (Buckner et al., 2011; Yeo et al., 2011). It has been revealed by distributed cerebellar networks that support the movement, attention, and limbic valence, as well as frontoparietal and default systems with topographic specificity mapping to the cerebellum (Habas et al., 2009; O’Reilly et al., 2010). To define the cerebellar networks, Yeo et al. (2011) proved 17 divergent networks possessing relatively stable parcellation solutions, in which the major regions of the cerebellar cortex are linked to cerebral networks. Baker et al. (2014) provided the names and cortical regions of the 17 networks in the Supplementary material. These networks contain the peripheral vision (N1), the central vision (N2, region Vermis VI), somato-motor A (N3, regions I-V and VIIb), somato-motor B (N4, region V), dorsal attention A (N5), dorsal attention B (N6, region VIIb-VIIIa), ventral attention (N7, regions VI and VIIIa), salience (N8, regions VI, Crus I-II, and VIIb), limbic (N9-10, white matter), control C (N11, region Crus I), control A (N12, regions VI and VIIb), control B (N13, regions Crus I and VIIb), auditory (N14), default C (N15, region X), default A (N16, regions IX, Vermis IX, Crus I, and Crus II), and default B (N17, region Crus I-II) network (Baker et al., 2014).

Several studies have confirmed that cerebro-cerebellar dysconnectivity is displayed in patients with SCH in association with somatomotor function (Chen et al., 2013; Shinn et al., 2015). Considering the modular architecture of cerebro-cerebellar circuitry, researchers have observed that patients with SCH showed a pattern of reduced functional connectivity (FC) between the cerebellum and a set of cerebral regions, such as the left middle temporal gyrus, right paracentral lobule, and right thalamus (Liu et al., 2011). Moreover, the dysconnection of the cerebellum in patients with SCH is found in the cingulo-opercular network, the right frontoparietal network, and the motor network (Chen et al., 2013). In SCH patients with auditory hallucinations, the main causal source is an occipital-cerebellar component compared with patients with SCH without auditory hallucinations and healthy controls (HCs) (de la Iglesia-Vaya et al., 2014). A recent study further has indicated that the left cerebellar posterior lobe is found to be negatively correlated with negative symptoms of patients with SCH (Yang et al., 2021). Therefore, the importance of assessing the organization and functional connection in the cerebellum is to better understand its role in SCH. However, the FC of cerebellar networks in SCH remains elusive.

Given the functional heterogeneity within the cerebellum, we hypothesized that cerebellar network connectivity would be aberrant in highly selective ways, exhibiting decreased SCH compared to HCs. In addition, we expected that cerebellar networks’ abnormalities are relative to positive and negative symptoms of patients with SCH. In this study, we first examined the FC of resting-state cerebellar networks in patients with first-episode SCH and HCs. Besides, we conducted a correlation analysis between the FC of cerebellar networks and positive and negative symptoms in patients with first-episode SCH. Finally, we established the classification model to provide relatively suitable features for patients with first-episode SCH concerning the cerebellar networks.

## Materials and methods

### Participants

A total of 45 patients with first-episode SCH without any prior treatment and 45 HCs matched for gender and age were recruited in the study. This study was approved by the Ethics Committee of Beijing Anding Hospital, Capital Medical University, and all protocols were carried out under the guidance of the Declaration of Helsinki. Informed consent was obtained from all participants before the study procedures. All participants were the Chinese Han people.

Patients were included if (a) they were diagnosed as SCH by two trained psychiatrists under diagnostic and statistical manual of mental disorders (DSM)-IV criteria; (b) they had not received any psychotropic medications (e.g., antipsychotics); (c) they were 16–45 years old and right-handed; (d) their highest educational attainment was higher than a secondary school; and (e) they are without any contraindications to MRI. Participants were excluded if they (a) had a current comorbid substance-use disorder (daily consumption of substances for at least 1 year) and (b) had a history of neurological disorders or a family history of hereditary neurological disorders. In addition, HCs were included if they were not diagnosed with any psychiatric diseases or other chronic/acute diseases (self-report). Psychiatric symptomatology was evaluated by using the Positive and Negative Syndrome Scale (PANSS) (Kay et al., 1987).

### MRI data acquisition

The MRI data were acquired with a 3.0 Tesla MRI scanner (Prisma 3.0; Siemens, Munich, Germany) in the Beijing Anding Hospital, Capital Medical University, China. Resting-state functional MRI was acquired with a single-shot, gradient-recalled echo-planar imaging sequence with the following parameters: repetition time = 2,000 ms, echo time = 30 ms, flip angle = 90°, matrix = 64 × 64, field of view = 200 mm × 200 mm, slice thickness = 3.5 mm, gap = 1 mm, 33 axial sections, and 240 volumes.

High-resolution brain structural images were acquired with a T1-weighted three-dimensional (3D) multi-echo magnetization-prepared rapid gradient-echo (MPRAGE) sequence (echo time: 3.39 ms, repetition time: 2,530 ms, slice thickness 1.3 mm, voxel size: 1 × 1 × 1 mm^3^, field of view (FOV): 256 × 256 mm^2^, and volume number: 128).

Prior to scanning, all participants were instructed to rest for 30 min. During scanning, they should remain still, keep their eyes closed, and not fall asleep.

### Image processing

All image processing was completed by the DPABISurf. As DPABISurf provides results in both surface and volume space, both surface-based and subcortical analyses can be performed. DPABISurf’s default preprocessing pipeline was used, which included converting the user-specified data into brain imaging data structure (BIDS), skull-stripping, spatial normalization, brain tissue segmentation, surface reconstruction for T1-weighted images and slice-timing correction, realignment, head-motion estimation, spatial registration, and smooth for functional images. The detailed methods were described in the article published by Esteban et al. (2019) and Yan et al. (2021). The head motion of participants was used to screen the quality control of images (mean FD_Jenkinson <2 mm).

We constructed a brain functional network for each subject according to the 17-network parcellation designed by Buckner et al. (2011). Detailed information on the parcellation is shown in Supplementary Table 1. Each node of the atlas was a sphere with a radius of 5 mm. The averaged BOLD signals across all voxels in the 17 regions of interest (ROIs) were extracted. The transformed *z*-scores of Pearson’s correlation coefficient (by Fisher’s *r*-to-*z* formula) of the BOLD signals were computed to define the FC for any pair of two ROIs; thus, a 17*17 matrix was conducted for each subject.

### Statistical analysis

Comparisons for clinical information were performed using an independent sample *t*-test (age) and a chi-square test (gender). The group-level network analysis was an independent sample *t*-test. We did not use any covariates in the group-level analysis for the *P*-values (age and gender) larger than 0.25 (Bursac et al., 2008). In addition, Cohen’s *f*^2^ value was used to describe the effect size. We used the false discovery rate (FDR) for multiple comparisons. The Pearson correlation analysis was applied to test the correlation between PANSS score and abnormal between-network FCs. Type I error was assumed to be 0.05.

### Feature selection and binary classification

We selected the significant intergroup differences between cerebellar networks’ FCs with correlation coefficient (*r* > 0.2 or *r* < −0.2) and participants’ age as features to distinguish patients with SCH from HCs. The linear support vector machine (LSVM) was conducted by using Python version 3.9.12 (anaconda version) with the Sklearn^1^ package. In LSVM, a well-defined sample was used to establish a decision boundary that could discriminate between categories and predict a new target subject’s membership in each group. In the training dataset, a 10-fold cross-validation approach was applied to train the best model. The receiver operating characteristic (ROC) curve and the area under the ROC curve were calculated based on the 10-fold validation results to quantify the performance of the model.

## Results

### Clinical information

A total of 90 participants (SCH: HC = 1:1) were analyzed without gender and age significant differences. The 45 patients with first-episode SCH had a 66.51 ± 14.634 PANSS score. Additionally, none were excluded for head motion or structural abnormalities in the brain. The details are shown in Table 1.

**TABLE 1:** Clinical information of participants.

	SCH (*N* = 45)	HC (*N* = 45)	*P*
Gender (Male/Female)	17/28	16/29	0.827
Age	23.0 (6.3)	23.3 (2.3)	0.823
PANSS			
Positive scores	18.2 (3.8)	–	–
Negative scores	17.2 (5.5)	–	–
General psychopathology scores	31.1 (6.6)	–	–
Total scores	66.5 (14.6)	–	–

SCH, schizophrenia; HC, healthy control; PANSS, Positive and Negative Syndrome Scale.

### Network analysis

**FIGURE 1:**
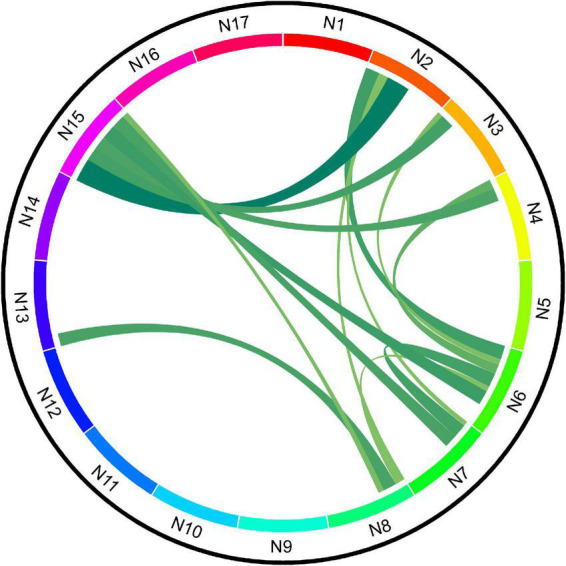
Schematic diagram of functional connectivity (FC) between cerebellar networks of schizophrenia (SCH) and healthy controls (HCs).

**FIGURE 2:**
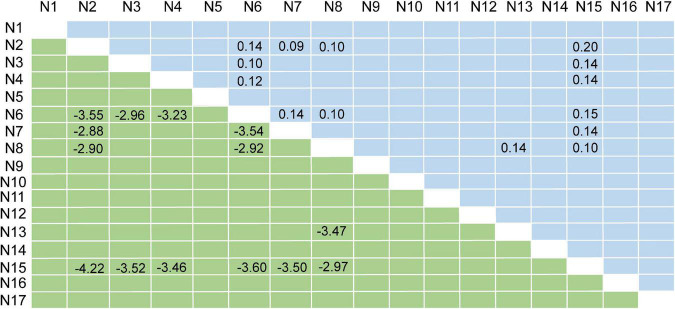
Results of network analysis. The lower triangle represents the *t*-values; the upper triangle represents Cohen’s *f*^2^ values.

Compared with HCs, patients with SCH demonstrated lower between-network FCs between 14 distinct cerebellar network pairs, namely, N2-N6, N2-N7, N2-N8, N2-N15, N3-N6, N3-N15, N4-N6, N4-N15, N6-N7, N6-N8, N6-N15, N7-N15, N8-N13, and N8-N15. The Circos of network analysis is shown in Figure 1. The detailed *t*-values (lower triangle) and Cohen’s *f*^2^ values (upper triangle) are shown in Figure 2. According to Cohen’s *f*^2^ value, the N2-N15 and N6-N15 had a medium effect size.

### Correlation analysis results

**FIGURE 3:**
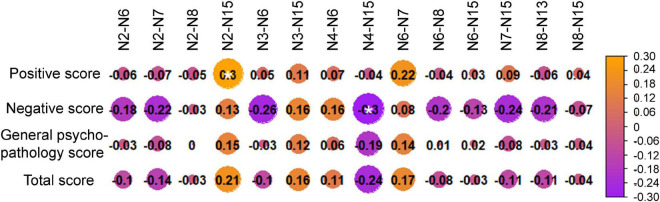
Correlation analysis results between Positive and Negative Syndrome Scale (PANSS) scores and altered between-network functional connectivity (FCs).

**FIGURE 4:**
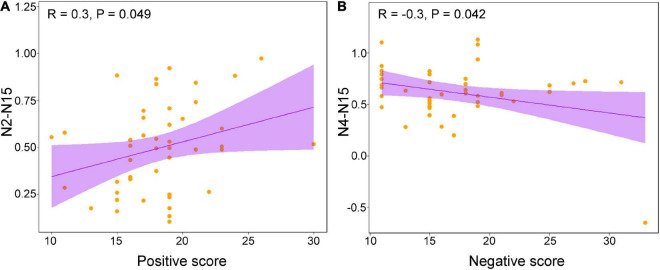
**(A)** The result of correlation analysis between positive score and N2-N15 and **(B)** the result of correlation analysis between negative score and N4-N15.

The correlation analysis between PANSS scores and altered between-network FCs showed that the N2-N15 was positively correlated with positive scores (Figures 3, 4A), and the N4-N15 was negatively correlated with negative scores (Figure 4B).

### Machine learning results

**FIGURE 5:**
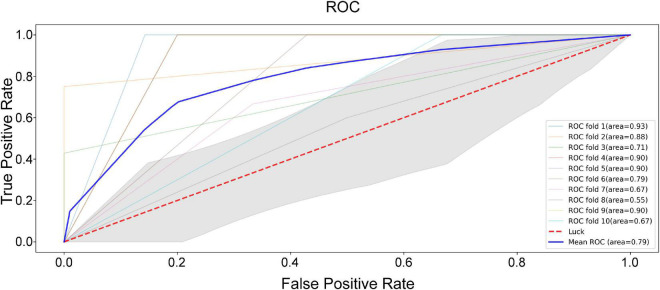
The area under curve (AUC) score of the linear support vector machine (LSVM) classification.

According to the correlation analysis, eight features were selected for LSVM analysis, namely, N2-N7 (*r* = −0.22 with negative scores), N2-N15 (*r* = 0.3 with positive scores; *r* = 0.21 with PANSS total scores), N3-N6 (*r* = −0.26 with negative scores), N4-N15 (*r* = −0.3 with positive scores; *r* = −0.24 with PANSS total scores), N6-N7 (*r* = 0.22 with positive scores), N6-N8 (*r* = −0.2 with negative scores), N7-N15 (*r* = −0.24 with negative scores), and N8-N14 (*r* = −0.21 with negative scores). In addition, the characteristics of these features between patients with SCH and HC are shown in Table 2. As shown in Figure 5, the best area under curve (AUC) score of the LSVM classification was 0.93, and the mean AUC score was 0.79.

**TABLE 2:** Characteristics of the eight network features for discriminating patients with schizophrenia (SCH) and healthy control (HC) subjects.

	SCH (*N* = 45)	HC (*N* = 45)	*P*
N2-N7	1.06 (0.30)	0.84 (0.43)	0.005
N2-N15	0.72 (0.26)	0.50 (0.24)	<0.001
N3-N6	0.88 (0.30)	0.70 (0.27)	0.004
N4-N15	0.81 (0.27)	0.61 (0.28)	0.001
N6-N7	1.28 (0.28)	1.09 (0.23)	0.001
N6-N8	1.04 (0.25)	0.87 (0.30)	0.004
N7-N15	0.90 (0.26)	0.68 (0.32)	0.001
N8-N13	1.09 (0.22)	0.93 (0.23)	0.001

SCH, schizophrenia; HC, healthy controls. Two-sample t-tests were used to compare the differences between two groups.

## Discussion

In this study, our findings revealed that the patients with first-episode SCH demonstrated lower between-network FCs between 14 distinct cerebellar network pairs, compared to the HCs. Notably, the between-network FC in N2-N15 was positively associated with positive symptom severity; meanwhile, N4-N15 was negatively associated with negative symptom severity. In addition, our LSVM analysis revealed a satisfactory classification accuracy (79%) of these decreased between-network FCs of cerebellar networks for correctly identifying patients with first-episode SCH.

In line with our hypothesis, we found that decreased between-network FCs of cerebellar networks are mainly involved in N2, N3, N4, N6, N7, N8, N13, and N15. The cerebellum is a complex region with many subregions dedicated to different functions associated with higher cortical areas (Andreasen and Pierson, 2008). Previously published neuroimaging studies have demonstrated that the cerebellum links to cerebral regions by the basal ganglia forming the cerebellar-cortical circuit (Duan et al., 2015). The 17 cerebellar networks were defined using the cerebro-cerebellar FC (Buckner et al., 2011).

The altered FCs of cerebellar networks in the current study belonging to the cerebellar motor and cognitive modules are mainly coupled with cortical somatosensory network (SMN), default mode network (DMN), salience network (SN), and dorsal attention network (DAN) (Balsters et al., 2014), which are associated with motor and cognitive functions. In accordance with our results, patients with first-episode SCH revealed decreased amplitude of low-frequency fluctuations in the right Crus I (belonging to the cerebellar cognitive module) (Guo et al., 2018) and decreased gray matter across motor and cognitive cerebellar modules (He et al., 2019). The reduced FC between the cerebellum and a set of cerebral regions, such as the left middle temporal gyrus, right paracentral lobule, and right thalamus, was observed in patients with SCH (Liu et al., 2011). Another previous study on patients with SCH also showed decreased cerebro-cerebellar FC in ventral attention network (N7), SN (N8), control A (N12), control B (N13), and DMN A (N16) (Shinn et al., 2015). Nevertheless, there was controversy in terms of FC changes in SCH. The increased FC in SCH was found between the cerebellum (lobule IX, lobule X, Crus I, and CrusII) and the ventral attention network, which was interpreted as the compensatory adaptation (Kim et al., 2020). To explain this controversy, Kim et al. (2020) proposed that functional efficiency, not the functional activity itself, was impaired between the cerebral network and cerebellum in SCH. Apart from this, the cerebellum has gained more attention in several psychiatric disorders, such as bipolar disorder, major depressive disorder, and generalized anxiety disorder (van Dun et al., 2022). Most studies have reported the abnormalities of FC between the cerebellum and the cerebral cortex in these psychiatric disorders (Phillips et al., 2015). However, the communication of cerebellar networks in these psychiatric disorders remains unclear. Thus, we focused on the FC between cerebellar networks in patients with SCH compared with HCs to explore the neural mechanism underlying the wide-ranging symptoms in SCH. Future studies are required to explore the cerebellar networks in other neuropsychiatric diseases to differentiate these disorders.

The network N2 is primarily associated with locomotion and body posture (Coffman et al., 2011). The network N15 includes the retrosplenial complex, parahippocampal complex, and ventral inferior parietal associated with the prefrontal area (Buckner et al., 2011). In the present study, we found a decreased between-network FC value of N2-N15 in patients with SCH compared to HCs. Furthermore, we observed that the SCH patients with severer positive symptoms showed less decreased between-network FC values of N2-N15, suggesting the underlying relationship between motor function and positive symptoms of SCH. Clinically, increasing evidence has shown that psychotic symptoms are accompanied by some motor impairments in patients with SCH (Gebhardt et al., 2008; Peralta and Cuesta, 2011). A recent study has illustrated that motor function is negatively correlated with positive symptoms (i.e., hallucinations and delusions) in SCH (Wang et al., 2020). Also, it has been found that motor dysfunction involves circuits linking with the cerebellum, thalamus, and basal ganglia (Andreasen et al., 1998). Neuroimaging studies have demonstrated that patients with SCH are characterized by abnormalities within the thalamic–prefrontal–cerebellar network (Woodward et al., 2012). Moreover, a recent study has exhibited that the FC between the thalamus and cerebellar is negatively associated with positive symptoms (Ferri et al., 2018). Significantly, patients with SCH manifest with lower connectivity between the thalamus and prefrontal cortex, while patients show stronger connectivity between the thalamus and motor/somatosensory regions (Woodward et al., 2012; Damaraju et al., 2014). Noteworthy, our findings showed that the between-network FC of N4-N15 yielded a trend for a negative correlation with the severity of negative symptoms. The N4 correlated with motor cortical regions contains central sulcus, secondary somatosensory, insula, and auditory contributing to the somatomotor map (Kelly and Strick, 2003; Krienen and Buckner, 2009). In line with our results, a recent study has revealed that the left cerebellar posterior lobe was negatively correlated with negative symptoms of patients with SCH (Yang et al., 2021). Another study also confirmed that the FC between the dorsolateral prefrontal cortex and the cerebellar network was negatively correlated with negative symptom severity in SCH (Brady et al., 2019). Based on accumulating evidence, the change in FC of the cerebellar network was correlated with positive and negative symptoms of patients with SCH (Andreasen and Pierson, 2008; Picard et al., 2008; Pappa and Dazzan, 2009; Abboud et al., 2017). Together, the cerebellar networks might play a critical role in modulating positive and negative symptoms for SCH. Despite our results identifying the relationships between cerebellar networks and symptoms, it may be obfuscated by common factors in psychiatric neuroimaging studies such as cognitive function, social function, and aerobic fitness. Future studies are required to investigate whether these mediating factors contribute to the cerebellar networks’ FCs and symptoms of SCH.

Nevertheless, there were some limitations to be concerned about. First, we did not gather motor and cognitive behavioral data from the recruited participants. Future studies are needed to assess the cognitive and motor function in SCH and the relationship between cerebellar networks. Second, some mediating factors might play a role in the correlation between cerebellar networks and symptoms of patients with SCH. Future research is required to probe whether several mediating factors contribute to the cerebellar networks’ FCs and symptoms of SCH, such as cognitive function, social function, and fitness. Third, we conducted a cross-sectional study to explore the data at a single point in time. A longitudinal study is needed to investigate the changes in cerebellar networks and symptoms from time to time. Fourth, we performed the LSVM learning to train the best model, thereby distinguishing SCH from HCs. The independent validation sample is required to test the model in the future. Moreover, other psychiatric disorders are also needed to investigate in the cerebellar networks to discriminate SCH from other psychiatric disorders.

In conclusion, abnormalities between-network FCs of cerebellar networks were observed in first-episode SCH correlating positive and negative symptoms. The classification result between patients with first-episode SCH and HCs indicated the possible neural mechanisms for the involvement of cerebellar networks in patients with first-episode SCH.

## Data availability statement

The original contributions presented in this study are included in the article. Further inquiries can be directed to the corresponding authors.

## Ethics statement

The studies involving human participants were reviewed and approved by the Ethics Committee of Beijing Anding Hospital, Capital Medical University. Written informed consent to participate in this study was provided by the participants or their legal guardian/next of kin. Written informed consent was obtained from the individual(s) for the publication of any potentially identifiable images or data included in this article.

## Author contributions

YN and HJ: conception and design. SF, LD, HaZ, and SL: data collection. HoZ and SZ: data analysis. SF, YN, and SZ: writing. SF: English-language revision. HJ, DW, and YN: revision. All authors contributed to the final version of the manuscript and approved the submitted version.

## Footnotes

1
https://scikit-learn.org/stable/
