# Supplementary material for: Altered functional connectivity of cerebellar networks in first-episode schizophrenia
Source: Front Cell Neurosci. 2022 Nov 11;16:1024192. doi: 10.3389/fncel.2022.1024192 (PMC9692071; doi:10.3389/fncel.2022.1024192)
Supplement: Supplementary file 1 [file Table_1.DOCX]

**TABLE S1** The information of 17-network cerellum parcellation

| Name | Cerebellar anatomical regions | No. of voxels | Network Name in Baker et al., 2014 (Baker et al., 2014) | Cerebral cortical region |
| --- | --- | --- | --- | --- |
| N1 | N/A | 0 | Visual peripheral | Striate, extrastriate |
| N2 | Vermis VI | 14 | Visual central | Striate, extrastriate |
| N3 | I-V, VIIb | 489 | Somatomotor A | Central sulcus, secondary somatosensory |
| N4 | V | 241 | Somatomotor B | Central sulcus, secondary somatosensory, insula, auditory |
| N5 | N/A | 0 | Dorsal attention A | Posterior temporal occipital, superior parietal, inferior parietal occipital |
| N6 | VIIb-VIIIa | 238 | Dorsal attention B | Posterior temporal, postcentral gyrus, frontal eye fields, precentral ventral frontal |
| N7 | VI, VIIIa | 750 | Ventral attention | Parietal operculum, medial parietal, medial frontal, precentral ventral frontal, insula, temporal, precentral frontal, posterior temporal |
| N8 | VI, Crus I-II, VIIb | 439 | Salience | Medial posterior prefrontal, ventral prefrontal, cingulate sulcus, inferior parietal, lateral prefrontal |
| N9-10 | White matter | 1024 | Limbic | Temporal pole, orbitofrontal |
| N11 | Crus I | 9 | Control C | Precuneus, posterior cingulate |
| N12 | VI, VIIb | 426 | Control A | Intraparietal sulcus, lateral prefrontal, lateral anterior prefrontal, inferior parietal, temporal, medial posterior prefrontal |
| N13 | Crus I, VIIb | 1154 | Control B | Lateral posterior prefrontal, lateral anterior prefrontal, inferior parietal, temporal, medial posterior prefrontal |
| N14 | N/A | 0 | Default D (Auditory) | Temporal cortex |
| N15 | X | 30 | Default C | Retrosplenial, parahippocampal complex, ventral inferior parietal |
| N16 | IX, Vermis IX, Crus I, Crus II | 799 | Defualt A | Medial prefrontal, posterior inferior parietal, posterior cingulate, dorsal prefrontal, obitofrontal, temporal |
| N17 | Crus I-II | 1387 | Default B | Dorsal prefrontal, temporal, anterior inferior parietal |
